# Low Peripheral T Follicular Helper Cells in Perinatally HIV-Infected Children Correlate With Advancing HIV Disease

**DOI:** 10.3389/fimmu.2018.01901

**Published:** 2018-08-24

**Authors:** Bret McCarty, Mussa Mwamzuka, Fatma Marshed, Matthew Generoso, Patricia Alvarez, Tiina Ilmet, Adam Kravietz, Aabid Ahmed, William Borkowsky, Derya Unutmaz, Alka Khaitan

**Affiliations:** ^1^Department of Pediatrics, Division of Infectious Diseases, New York University School of Medicine, New York, NY, United States; ^2^Bomu Hospital, Mombasa, Kenya; ^3^Jackson Laboratory for Genomic Medicine, Farmington, CT, United States; ^4^Department of Microbiology, New York University School of Medicine, New York, NY, United States

**Keywords:** T follicular helper cells, HIV, children, immune activation, B cells, T follicular cytotoxic cells

## Abstract

**Background:**

T follicular helper (Tfh) cells are crucial for B cell differentiation and antigen-specific antibody production. Dysregulation of Tfh-mediated B cell help weakens B cell responses in HIV infection. Moreover, Tfh cells in the lymph node and peripheral blood comprise a significant portion of the latent HIV reservoir. There is limited data on the effects of perinatal HIV infection on Tfh cells in children. We examined peripheral Tfh (pTfh) cell frequencies and phenotype in HIV-infected children and their associations with disease progression, immune activation, and B cell differentiation.

**Methods:**

In a Kenyan cohort of 76 perinatally HIV-infected children, comprised of 43 treatment-naïve (ART−) and 33 on antiretroviral therapy (ART+), and 42 healthy controls (HIV−), we identified memory pTfh cells, T cell activation markers, and B cell differentiation states using multi-parameter flow cytometry. Soluble CD163 and intestinal fatty acid-binding protein plasma levels were quantified by ELISA.

**Results:**

ART− children had reduced levels of pTfh cells compared with HIV− children that increased with antiretroviral therapy. HIV+ children had higher programmed cell death protein 1 (PD-1) expression on pTfh cells, regardless of treatment status. Low memory pTfh cells with elevated PD-1 levels correlated with advancing HIV disease status, indicated by increasing HIV viral loads and T cell and monocyte activation, and decreasing %CD4 and CD4:CD8 ratios. Antiretroviral treatment, particularly when started at younger ages, restored pTfh cell frequency and eliminated correlations with disease progression, but failed to lower PD-1 levels on pTfh cells and their associations with CD4 T cell percentages and activation. Altered B cell subsets, with decreased naïve and resting memory B cells and increased activated and tissue-like memory B cells in HIV+ children, correlated with low memory pTfh cell frequencies. Last, HIV+ children had decreased proportions of CXCR5+ CD8 T cells that associated with low %CD4 and CD4:CD8 ratios.

**Conclusion:**

Low memory pTfh cell frequencies with high PD-1 expression in HIV+ children correlate with worsening disease status and an activated and differentiated B cell profile. This perturbed memory pTfh cell population may contribute to weak vaccine and HIV-specific antibody responses in HIV+ children. Restoring Tfh cell capacity may be important for novel pediatric HIV cure and vaccine strategies.

## Introduction

T follicular helper (Tfh) cells are a recently described CD4 T cell subset that links the adaptive and humoral immune systems. These cells are identified by expression of chemokine receptor CXCR5 that directs their migration to B cell follicles in response to CXCL13. Once localized in B cell follicles, Tfh cells form germinal centers (GCs) (1). Their differentiation and functions are regulated by B-cell lymphoma 6 (Bcl-6) (2). Tfh cells stimulate B cell differentiation to plasma and memory B cells (3) and are critical for antigen-specific antibody production, class switching, and B cell memory differentiation (4). During natural infections or after vaccinations, Tfh cell interactions with B cells mediate high affinity class-switched antibody production and B cell memory development (3). Tfh cells exert effector functions by secretion of IL-21 in addition to small levels of Th1 and Th2 cytokines IFNγ and IL-4 (4, 5). Dysfunctional Tfh cells can result in autoantibodies and have been associated with autoimmune diseases such as rheumatoid arthritis and systemic lupus erythematosus (6–10).

A small portion of CD4 T cells closely resembling tissue-resident Tfh cells are found in the peripheral blood (10–12). These peripheral Tfh (pTfh) cells also provide B cell help, but require secondary signals that include CD40L and inducible T-cell costimulator (ICOS) interactions and IL-21 secretion from B cells (11). Phenotypically, pTfh cells differ from lymphoid Tfh cells. Bcl-6 is downregulated in circulating CXCR5+ CD4 T cells, and thus fails to identify pTfh cells (10, 11). Second, while programmed cell death protein 1 (PD-1) is constitutively expressed on lymphoid Tfh cells, in the periphery, PD-1 is variably expressed, with low levels on resting pTfh cells and high levels on activated pTfh cells (12–14).

T follicular helper cells are critical for clearance of acute and chronic viral infections and effective virus-specific antibody production. In HIV vaccine trials, improved humoral responses occurred in subjects with expanded HIV-specific IL-21+ pTfh cells (14). Moreover, certain Tfh cell subsets correlate with the development of broadly neutralizing HIV antibodies (13, 15). Indeed, Tfh cells are being investigated for novel HIV vaccine strategies (11, 13, 15). Studies of HIV-infected adults demonstrate that circulating Tfh cells are decreased while lymphoid Tfh cells are paradoxically expanded, functionally impaired, and preferentially infected with replication-competent HIV (14, 16–20). Most importantly, Tfh cells in peripheral blood and lymph nodes (LNs) comprise a major compartment of the latent HIV reservoir (14, 21). Thus, Tfh cells may have both beneficial and pathologic roles during HIV infection—they are critical for HIV-specific humoral responses yet are also selective targets of HIV infection and enable HIV persistence in latent reservoirs.

Both lymphoid and circulating Tfh cells have impaired function in HIV+ adults, which may contribute to weakened responses to vaccines (16, 20, 22, 23). The potential consequences of muted antibody responses are magnified in children with perinatal HIV infection during routine childhood vaccinations. However, few studies have examined pTfh cells in children (18–20, 24). We evaluated pTfh cell frequencies and phenotype in children with perinatal HIV infection and their associations with HIV disease progression and B cell subsets. We found decreased pTfh cell levels in untreated HIV+ children compared with HIV negative children, which failed to normalize within 1 year of antiretroviral treatment. pTfh cells had elevated PD-1 expression in HIV+ children regardless of treatment status. Low pTfh cell frequencies with high PD-1 expression correlated with HIV disease progression and an activate/differentiated B cell distribution. Finally, CXCR5+ memory CD8 T cells were depleted in HIV+ children.

## Materials and Methods

### Participants

Ethical approval for this study was obtained from New York University (10-02586) and Kenyatta National Hospital/University of Nairobi (P283/07/2011). Written informed consent and verbal assent when appropriate were obtained from all participants and/or parents. We enrolled a total of 76 perinatally infected HIV+ and 42 HIV negative-unexposed children (HIV−) aged 5–18 years old from Bomu Hospital in Mombasa, Kenya between 2011 and 2012. HIV+ children included 43 antiretroviral therapy naïve (ART−) and 33 HIV+ children on antiretroviral treatment for at least 6 months with viral load less than or equal to 5,000 copies/mL (ART+). Treatment timing and duration for ART+ subjects is shown in Table S1 in Supplementary Material. Individuals with a recent acute illness, active *Mycobacterium tuberculosis* or malaria infection, or pregnancy within one year were ineligible for study entry.

Plasma and peripheral blood mononuclear cells (PBMCs) were isolated from peripheral blood by centrifugation and Ficoll-Hypaque (GE Healthcare) density gradient centrifugation then cryopreserved in −80°C and liquid nitrogen, respectively. HIV RNA PCR was performed on diluted plasma samples with Roche, COBAS^®^ AmpliPrep/COBAS^®^ TaqMan^®^HIV-1 Test, version 2.0 (limit of detection 110 copies/mL).

HIV−, ART−, and ART+ were matched for age and sex (Table 1). Median CD4% in HIV− children was 38 (IQR 33–42). ART− had median CD4% of 24 (IQR 13–28) and HIV viral load of 4.8 (IQR 4.2–5.2) log copies/mL. ART+ had median CD4% and HIV viral load of 32 (IQR 27–41) and 2 (IQR 2–2) log copies/mL, respectively (Table 1).

**Table 1:** Subject characteristics.

	HIV−	ART−	ART+	*p*
*n*	42	43	33	
Age (years)^d^	11 (9–13)	11 (8–14)	12 (8–13)	NS^a^
Female	17 (41%)	23 (54%)	19 (58%)	NS^b^
%CD4^d^	38 (33–42)	24 (13–28)	32 (27–41)	*p* < 0.0001^a^
Absolute CD4 count^d^	923 (739–1,247)	583 (352–812)	962 (750–1,228)	*p* < 0.0001^a^
HIV log copies/mL^d^		4.8 (4.2–5.2)	2 (2–2)	*p* < 0.0001^c^

*^a^Kruskal–Wallis test*.

*^b^Chi squared test*.

*^c^Mann–Whitney test*.

*^d^Median values with upper and lower quartile range*.

### Flow Cytometric Studies

Cryopreserved PBMCs were evaluated by flow cytometry with fluorescent-conjugated antibodies to CD3, CD4, CD8, CD45RO, CCR7, CXCR5, PD-1, CD38, HLA-DR, CD19, CD21, CD27, and β7. Cells were stained at 4°C for 30 min in PBS buffer containing 2% FCS and 0.1% sodium azide. Stained cells were analyzed using LSRII flow cytometer (BD Bioscience) and Flow Jo software (Tree Star). Singlet lymphocytes were gated based on forward and side scatter properties.

### Plasma sCD163 and Intestinal Fatty Acid-Binding Protein (I-FABP)

Plasma levels of sCD163 and I-FABP were quantified by ELISA assay using Human sCD163 and I-FABP Duoset kits (R&D Systems) per the manufacturer’s instructions. Plasma samples were diluted 1:100 for sCD163 and 1:1,500 for I-FABP assays based on plasma titration studies to achieve levels within the range of the standard curve concentrations provided in the commercial ELISA kit according to the manufacturer’s recommendation. Each test was performed in duplicate with results reported as the average of duplicate results.

### Statistics

All statistical analyses were performed using GraphPad Prism software. For comparison of multiple groups of subjects, the Kruskal–Wallis test was performed, followed by the two-stage linear step-up procedure of Benjamini, Krieger, and Yekutieli to correct for multiple comparisons by controlling the false discovery rate. Multiple time points were evaluated with Wilcoxon matched-pairs signed-rank test. Correlations were assessed with the Spearman rank test. Threshold of significance for all tests was less than 0.05.

## Results

### Memory pTfh Cells Are Decreased in Untreated HIV+ Children and Correlate With Disease Progression

**Figure 1:**
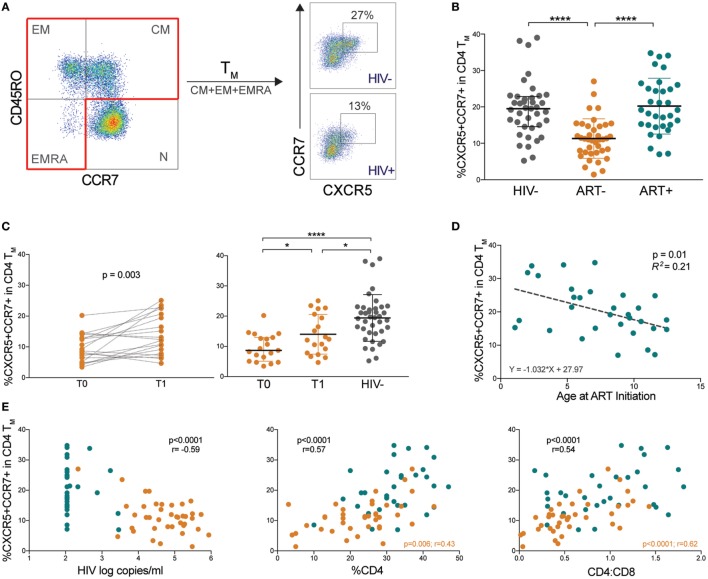
Memory peripheral Tfh (pTfh) cells are decreased in untreated HIV+ children and correlate with disease progression. **(A)** FACS plots showing representative gating to identify memory pTfh cells in an HIV− and HIV+ subject. CD4 memory subsets were identified by CD45RO and CCR7 expression. Total memory CD4 T cells (CD4 T_M_) were Boolean-gated as the sum of T_CM_, T_EM_, and T_EMRA_ populations. Memory pTfh cells are identified as CXCR5+ CCR7+ cells within CD4 T_M_. **(B)** The proportion of CXCR5+ CCR7+ cells in CD4 T_M_ in HIV−, ART−, and ART+ children. **(C)** Memory pTfh cell frequencies in ART− subjects at before (T0) and ~12 months after treatment (T1) is shown. The second plot shows memory pTfh cell levels in ART− subjects at T0 and T1 compared with HIV− subjects. **(D)** Linear regression plot of memory pTfh cells vs. age at ART initiation in ART+ children. **(E)** Correlations between memory pTfh cell frequencies and HIV log copies/mL, %CD4+ T cells, and CD4:CD8 ratios in HIV+ children (ART− in orange and ART+ in blue). Significant *p* values are shown for statistical analysis of HIV+ (black), ART− (orange), and ART+ (blue) groups (****p < 0.0001, ***p < 0.001, **p < 0.01, and *p < 0.05).

We identified pTfh cells by CXCR5 co-expressed with LN homing receptor CCR7 within memory CD4 T cells as described (25). Memory CD4 T cells (T_M_) were identified as the sum of central (T_CM_, CD45RO+CCR7+), effector (T_EM_, CD45RO+ CCR7−), and RA+ effector (T_EMRA_, CD45RO−CCR7−) subsets (Figure 1A). CXCR5+CCR7+ CD4 T_M_ (memory pTfh) levels were lower in ART− children compared with HIV− and ART+ (*p* < 0.0001; Figure 1B), even when adjusted for age as a potential confounder (Table S2 in Supplementary Material).

In a subset of ART− children who began antiretroviral therapy memory pTfh cell frequencies increased after ~12 months of treatment (*p* = 0.003; Figure 1C). However, these levels remained lower than HIV− children (*p* = 0.02; Figure 1C). In ART+ children, treatment initiation at an earlier age predicted preserved CXCR5+CCR7+ CD4 T_M_ (*p* = 0.01, *R*^2^ = 0.21; Figure 1D). To determine the clinical relevance of decreased pTfh cell frequencies, we examined correlations between memory pTfh cells and clinical markers of HIV disease progression. In HIV+ subjects, memory pTfh cell levels correlated inversely with HIV viral load (*p* < 0.0001, *r* = −0.59) and directly with %CD4 (*p* < 0.0001, *r* = 0.57) and CD4:CD8 ratios (*p* < 0.0001, *r* = 0.54; Figure 1E). In separate analyses of ART− and ART+ children, memory pTfh frequencies correlated with %CD4 (*p* = 0.006, *r* = 0.43) and CD4:CD8 ratios (*p* < 0.0001, *r* = 0.62) in ART− but not in ART+ children. These correlations were not present in HIV− subjects (Figure S1A in Supplementary Material).

### Low Memory pTfh Cell Frequencies Correlate With Immune Activation and Gut Mucosal Disruption in HIV+ Children

**Figure 2:**
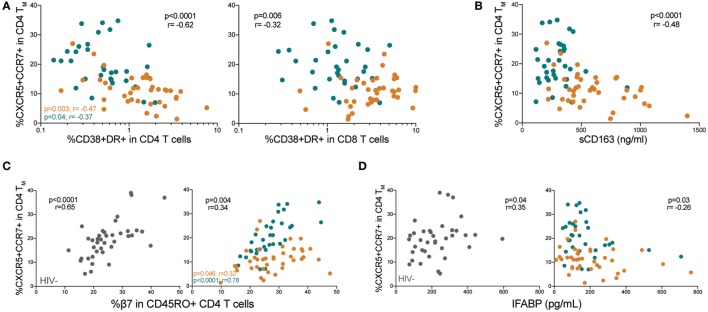
Low memory peripheral Tfh (pTfh) cell frequencies correlate with immune activation and gut mucosal disruption in HIV+ children. **(A)** Correlations between memory pTfh cell frequencies and CD38+ HLA-DR+ CD4 and CD8 T cells in HIV+ children (ART− in orange and ART+ in blue). **(B)** Correlation between memory pTfh cells and plasma soluble CD163 (sCD163) levels in HIV+ children. **(C)** Correlation between memory pTfh cell levels and **(C)** β7 integrin and **(D)** intestinal fatty acid-binding protein (I-FABP) in HIV− (gray circles) and HIV+ children. Significant *p* values are shown for statistical analysis of HIV+ (black), ART− (orange), and ART+ (blue) groups.

We next determined whether memory pTfh cell levels correlated with immune activation markers CD38 and HLA-DR, which are strong predictors of HIV disease progression (26). Memory pTfh cell frequencies inversely correlated with CD38+HLA-DR+ CD4 (*p* < 0.0001, *r* = −0.62) in HIV+ children and in separate analysis of ART− (*p* = 0.003, *r* = −0.47) and ART+ subjects (*p* = 0.04, *r* = −0.37; Figure 2A). Memory pTfh cell frequencies also negatively correlated with CD38+HLA-DR+ CD8 T cells (*p* = 0.006, *r* = −0.32) and monocyte activation, measured by plasma sCD163 concentrations in HIV+ children (*p* < 0.0001, *r* = −0.48), but not when divided into ART− and ART+ groups (Figures 2A,B). There were no significant correlations between pTfh cell frequencies and T cell or monocyte activation in HIV− children (Figures S1B,C in Supplementary Material).

Chronic inflammation in HIV is driven largely by a compromise of the gut mucosa, where the majority of CD4 T cells reside (27). In mouse models, Tfh cells have been shown to play a unique role in maintaining healthy gut homeostasis (28). To study a potential relationship between memory pTfh cells and the gut mucosa in humans, we evaluated correlations with two key gut-related proteins: β7 integrin and I-FABP. β7 is a subunit of the gut-homing receptor and HIV co-receptor α_4_β_7_ (29). I-FABP is expressed in epithelial cells of the small intestine and is released into the circulation following intestinal mucosal damage (30). Memory pTfh cell levels directly correlated with β7+CD45RO+ CD4 T cell frequencies, in both HIV− (*p* < 0.0001, *r* = 0.65) and HIV+ children (*p* = 0.004, *r* = 0.34; Figure 2C). ART+ children had a stronger correlation between pTfh cell frequency and β7 expression in memory CD4 T cells (*p* < 0.0001, *r* = 0.78) compared with ART− children (*p* = 0.046, *r* = 0.32; Figure 2C). There was a direct correlation between memory pTfh cell frequencies and I-FABP levels (*p* = 0.04, *r* = 0.35) in HIV− children, and an indirect correlation in HIV+ children (*p* = 0.03, *r* = −0.26; Figure 2D) but not separately in ART− and ART+ groups.

### Memory pTfh Cells in HIV+ Children Express High PD-1 Levels That Correlate With Disease Progression

**Figure 3:**
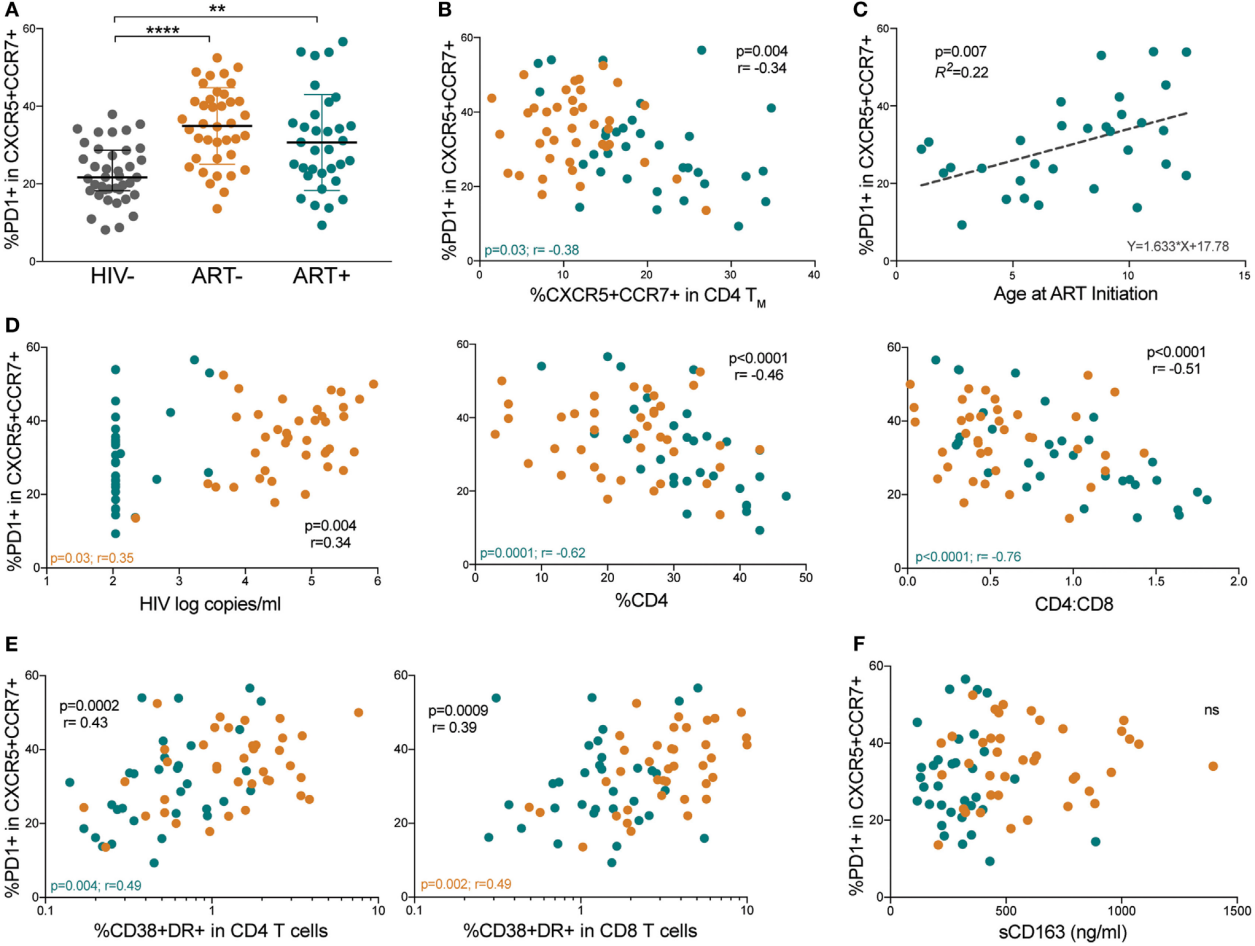
Memory peripheral Tfh (pTfh) cells in HIV+ children express high programmed cell death protein 1 (PD-1) levels that correlate with disease progression. **(A)** Comparison between PD-1 expression memory pTfh cells of HIV−, ART−, and ART+ children. **(B)** Correlation between the frequency of total memory pTfh cells and their PD-1 expression. **(C)** Linear regression plot of PD-1 expression on memory pTfh cells vs. age at ART initiation in ART+ subjects. **(D)** Correlations between the frequency of PD-1+ memory pTfh cells and HIV log copies/mL, %CD4+ T cells, and CD4:CD8 ratios in HIV+ children (ART− in orange and ART+ in blue). Correlations between PD-1 expression on memory pTfh and **(E)** CD38+ HLA-DR+ CD4 and CD8 T cells and **(F)** plasma sCD163 levels. Significant *p* values are shown for statistical analysis of HIV+ (black), ART− (orange), and ART+ (blue) groups (****p < 0.0001, ***p < 0.001, **p < 0.01, and *p < 0.05).

We next evaluated PD-1 expression on memory pTfh cells (gating strategy shown in Figure S2A in Supplementary Material). Both ART− and ART+ subjects had higher PD-1 expression on memory pTfh cells compared with HIV− children (*p* < 0.0001 and *p* = 0.003, respectively; Figure 3A). PD-1 levels on memory pTfh cells negatively correlated with pTfh cell frequency in HIV+ (*p* = 0.004, *r* = −0.34) and ART+ children (*p* = 0.03, *r* = −0.38) but not in ART− children (Figure 3B). In ART+ children, earlier age at ART initiation predicted lower PD-1 expression on memory pTfh cells (*p* = 0.007, *R*^2^ = 0.22; Figure 3C). PD-1+ memory pTfh cell frequencies correlated directly with HIV viral load (*p* = 0.004, *r* = 0.34), and inversely with %CD4 (*p* < 0.0001, *r* = −0.46) and CD4:CD8 ratios in HIV+ children (*p* < 0.0001, *r* = −0.51; Figure 3D) but not in HIV− children (Figure S2B in Supplementary Material). PD-1+ expression on memory pTfh cell correlated directly with CD38+HLA-DR+ CD4 (*p* = 0.0002, *r* = 0.43) and CD8 T cells (*p* = 0.0009, *r* = 0.39; Figure 3E) but did not correlate with plasma sCD163 levels (Figure 3F) in HIV+ children. In ART− children, PD-1 expression on pTfh cells significantly correlated with HIV viral load (*p* = 0.03, *r* = 0.35, Figure 2D) and CD38+HLA-DR+ CD8 T cells (*p* = 0.002, *r* = 0.49; Figure 3E). In ART+ children, PD-1 expression correlated inversely with %CD4 (*p* = 0.0001, *r* = −0.62) and CD4:CD8 ratios (*p* < 0.0001, *r* = −0.76; Figure 3D). There were no significant correlations between PD-1 expression on pTfh cells and %CD4 or immune activation markers in HIV− children (Figures S2C,D in Supplementary Material).

### Differentiated B Cell Populations Correlate With Low Memory pTfh Cells Expressing High PD-1 Levels

**Figure 4:**
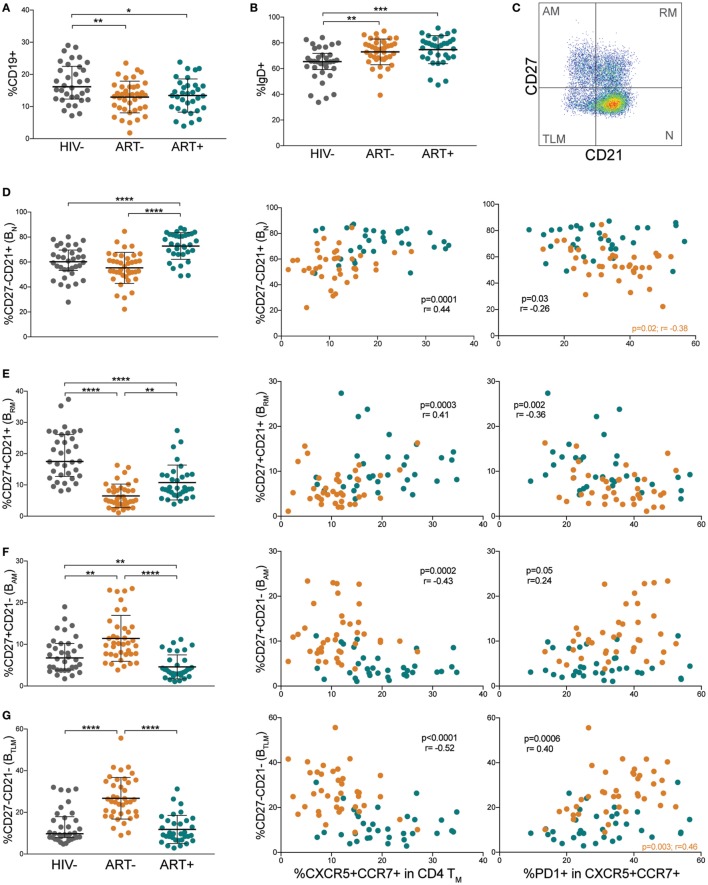
Differentiated B cell populations correlate with low memory peripheral Tfh (pTfh) cells expressing high programmed cell death protein 1 (PD-1) levels. Comparisons of **(A)** total B cell and **(B)** IgD+ B cell levels in HIV−, ART−, and ART+ children. **(C)** FACS plot showing representative gating to identify B cell differentiation subsets by CD21 and CD27. Plot shown is gated on CD19+ lymphocytes. Comparisons between **(D)** CD27−CD21+ naïve (B_N_), **(E)** CD27+ CD21+ resting memory (B_RM_), **(F)** CD27+ CD21− activated memory (B_AM_), and **(G)** CD27−CD21− tissue-like memory (B_TLM_) B cell subsets in HIV−, ART−, and ART+ and their correlations with total and PD-1+ memory pTfh cell frequencies in ART− (orange) and ART+ (blue) children. Significant *p* values are shown for statistical analysis of HIV+ (black), ART− (orange), and ART+ (blue) groups (****p < 0.0001, ***p < 0.001, **p < 0.01, and *p < 0.05).

Because Tfh cells are intricately linked to B cell differentiation, we evaluated B cell populations and their associations with memory pTfh cell frequencies and PD-1 expression. Total B cell frequencies were decreased in both ART− and ART+ compared with HIV− children (*p* = 0.005 and *p* = 0.02, respectively; Figure 4A). IgD expression on B cells was increased in ART− and ART+ children compared with HIV− children (*p* = 0.002 and *p* = 0.0001, respectively; Figure 4B), indicating muted class switching. We further sub-classified B cells into differentiation states of naïve mature (B_N_, CD21+CD27−), resting memory (B_RM_, CD21+CD27+), activated memory (B_AM_, CD21−CD27+), and exhausted/tissue-like memory (B_TLM_, CD21−CD27−; Figure 4C) subsets. ART+ had increased B_N_ frequencies compared with HIV− and ART− (*p* < 0.0001; Figure 4D). ART− and ART+ had decreased B_RM_ levels compared with HIV− (*p* < 0.0001; Figure 4E). B_RM_ levels were higher in ART+ compared with ART− children (*p* = 0.002; Figure 4E). ART− had elevated B_AM_ and B_TLM_ cell frequencies compared with HIV− (*p* = 0.003 and *p* < 0.0001) and ART+ (*p* < 0.0001; Figures 4F,G). ART+ had lower B_AM_ frequencies compared with HIV− (*p* = 0.003; Figure 4F).

We next examined associations between B cell differentiation states and memory pTfh cell frequencies. In HIV+ subjects, memory pTfh cell levels correlated directly with B_N_ (*p* = 0.0001, *r* = 0.44; Figure 4D) and B_RM_ frequencies (*p* = 0.0003, *r* = 0.41; Figure 4E), and inversely with B_AM_ (*p* = 0.0002, *r* = −0.43; Figure 4F) and B_TLM_ frequencies (*p* < 0.0001, *r* = −0.52; Figure 4G). Last, in HIV+ subjects, PD-1 expression on memory pTfh cells correlated inversely with B_N_ (*p* = 0.03, *r* = −0.26; Figure 4D) and B_RM_ (*p* = 0.002, *r* = −0.36; Figure 4E), and directly with B_AM_ (*p* = 0.046, *r* = 0.24; Figure 4F) and B_TLM_ levels (*p* = 0.0006, *r* = 0.40; Figure 4G). These correlations were insignificant when separated into ART− and ART+ subjects except PD-1 expression on pTfh cells in ART− children inversely correlated with B_N_ frequency (*p* = 0.02, *r* = −0.38; Figure 4D) and directly with B_TLM_ frequency (*p* = 0.003, *r* = 0.46; Figure 4G). Memory pTfh cells and their PD-1 expression did not correlate with any B cell subpopulations in HIV− subjects (Figures S3A–D in Supplementary Material).

### HIV+ Children Have Low CXCR5+ Memory CD8 T Cells With Elevated PD-1 Expression

**Figure 5:**
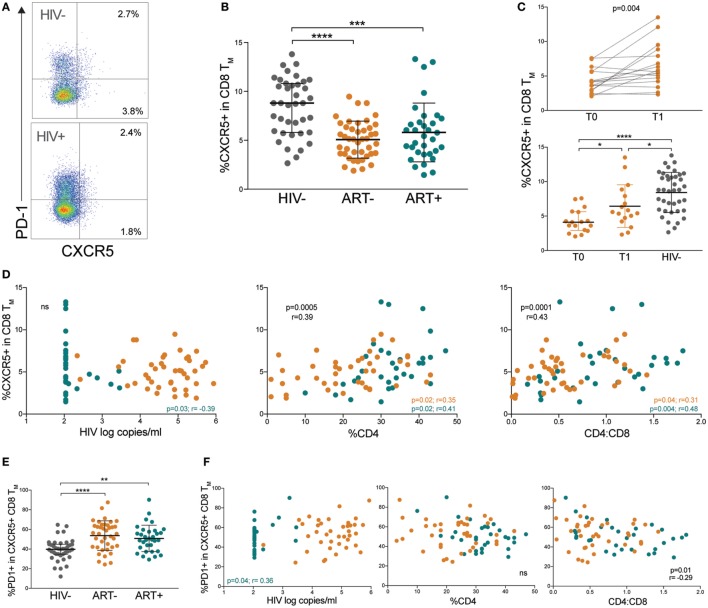
HIV+ children have low CXCR5+ memory CD8 T cells with elevated programmed cell death protein 1 (PD-1). **(A)** FACS plots showing representative gating to identify memory pTfc cells as the total CXCR5+ population in memory CD8 T cells and PD-1 expression in an HIV− and HIV+ subject. Plots shown were gated within total memory CD8 T cells. **(B)** Comparison of CXCR5+ cells within total CD8+ memory T cells in HIV−, ART−, and ART+ children. **(C)** Prospective analysis of memory pTfc in ART− subjects before (T0) and ~12 months after antiretroviral treatment (T1). The second plot compares memory pTfc cell frequencies between ART− subjects at T0 and T1 and HIV− subjects. **(D)** Correlations between memory pTfc cell percentages and HIV log copies/mL, %CD4+ T cells, and CD4:CD8 ratios in HIV+ children (ART− in orange and ART+ in blue). **(E)** PD-1 expression on memory pTfc cells in HIV−, ART−, and ART+ subjects. **(F)** Correlation between PD-1+ memory pTfc cell frequencies and HIV log copies/mL, %CD4+ T cells, and CD4:CD8 ratios in HIV+ children. Significant *p* values are shown for statistical analysis of HIV+ (black), ART− (orange), and ART+ (blue) groups (****p < 0.0001, ***p < 0.001, **p < 0.01, and *p < 0.05).

Recently, a CD8 T cell counterpart to Tfh cells, defined as follicular cytotoxic T (Tfc) cells, was found to home to B cell follicles *via* a similar CXCR5-dependent mechanism (31). In the B cell follicle, Tfc cells control viral infection by killing infected Tfh cells and B cells (32, 33). We identified pTfc cells by CXCR5 expression in memory CD8 T cells (Figure 5A). CXCR5+ CD8 T_M_ (memory pTfc) cell frequencies were significantly lower in both ART− and ART+ children compared with HIV− (*p* < 0.0001 and *p* = 0.0002, respectively; Figure 5B) even in multivariate analysis adjusting for age (Table S2 in Supplementary Material). In ART− subjects, memory pTfc cell frequencies increased after ~12 months of antiretroviral treatment (*p* = 0.004), but remained significantly lower than HIV− children (*p* = 0.03; Figure 5C).

Next, we determined whether memory pTfc cell levels correlated with clinical markers of HIV disease progression and immune activation. In HIV+ subjects, there was no significant correlation between memory pTfc cell frequencies and HIV viral load. Memory pTfc cells directly correlated with %CD4 (*p* = 0.0005, *r* = 0.39) and CD4:CD8 ratios (*p* = 0.0001, *r* = 0.43) in HIV+ subjects even when divided into ART− and ART+ groups (Figure 5D). In HIV− subjects, memory pTfc cell frequencies also correlated with CD4 percentages and CD4:CD8 ratios (%CD4: *p* = 0.02, *r* = 0.37; CD4:CD8: *p* = 0.02, *r* = 0.37; Figure S4A in Supplementary Material). We also examined PD-1 expression on memory pTfc cells and found that memory pTfc cells in HIV+ children expressed higher levels of PD-1 compared with HIV− children (ART−: *p* < 0.0001, ART+: *p* = 0.001; Figure 5E). In HIV+ children, PD-1 expression on memory pTfc cells inversely correlated with CD4:CD8 ratios (*p* = 0.01, *r* = −0.29), but did not correlate with %CD4 or viral load (Figure 5F). In ART+ subjects, HIV viremia correlated negatively with memory pTfc cell frequency (*p* = 0.03, *r* = −0.39; Figure 1D) and directly with PD-1 levels on memory pTfc cells (*p* = 0.04, *r* = 0.36; Figure 5F). Memory pTfc cell frequencies and their PD-1 expression did not correlate with CD38+HLA-DR+ CD4 or CD8 T cells or sCD163 plasma levels in HIV+ or HIV− children with the exception of a direct correlation between PD-1+CXCR5+ CD8 T_M_ and CD38+ HLA-DR+ CD8 T cells in HIV+ children (Figures S4B,C in Supplementary Material).

## Discussion

We demonstrated that untreated children with perinatal HIV infection aged 5–18 years have significantly lower memory pTfh cell frequencies compared with HIV-negative children. Low memory pTfh levels increased with antiretroviral treatment but failed to normalize. In ART+ children, treatment initiation at younger ages predicted preserved pTfh levels. HIV+ children had higher PD-1 expression on pTfh cells regardless of treatment status. Furthermore, lower memory pTfh cells with increased PD-1 expression correlated with worsening HIV disease and an activated and differentiated B cell profile in HIV+ children. Last, HIV+ children have decreased proportions of memory pTfc cells with high PD-1 expression.

In our cohort, low memory pTfh cell frequencies correlated with decreased %CD4 and CD4:CD8 ratios and increased HIV viral load, which may reflect preferential infection of Tfh cells by HIV (14). Muema et al. similarly reported that CXCR5+ CD4 T cells correlated with %CD4 in HIV+ children, yet Bamford et al. failed to find any correlation between pTfh cell frequencies and clinical variables in children. This difference may relate to treatment status, as in our cohort, where these correlations were no longer significant when limited to only ART+ children. While the mechanism of pTfh cell depletion in HIV is not well understood, it is possible that like tissue-resident Tfh cells, pTfh cells are preferentially HIV-infected and killed. It is also plausible that pTfh cells in HIV+ subjects are ill-maintained as a result of impaired crosstalk with B cells, as Tfh and B cells need constant communication *via* co-stimulatory signals to maintain a homeostatic and healthy immune system (2, 16). Alternatively, Tfh cells may be chronically activated and subsequently sequestered in the LNs, depleting levels in the peripheral blood as shown in SIV (34). While Pallikkuth et al. reported a reversible depletion of CXCR5+ cells within T_CM_ in adults with HIV (21), the recovery of pTfh cells with antiretroviral therapy in children is previously unreported. Treatment initiation raised pTfh cell frequencies in ART− subjects, but failed to restore them to normal levels within one year. However, our ART+ subjects had pTfh cell frequencies similar to HIV− children. This may be due to longer duration of antiretroviral treatment or younger age at treatment initiation, which predicted higher pTfh levels. We speculate that with early antiretroviral treatment, HIV+ children may be able to recover memory pTfh cells, in accordance with the recent recommendation from the World Health Organization to start treatment at the time of HIV diagnosis (35).

Programmed cell death protein 1 is a classic marker of functional Tfh cells in the lymphoid tissue of healthy adults and children, where PD-1/PD-L1 interactions in the GC are crucial for plasma cell differentiation (36). However, there is some uncertainty as to the role of PD-1 on circulating Tfh cells. PD-1 is a co-stimulatory molecule; it is absent on quiescent pTfh cells, expressed on follicular regulatory T (Tfr) cells, and co-expressed with ICOS on activated pTfh cells (37). Interestingly, we demonstrated PD-1 expression on memory pTfh cells was elevated in all HIV+ children regardless of treatment status, and corresponded with worsening HIV disease progression. Moreover, in ART+ children, PD-1 expression correlated with lower CD4 percentages and CD4:CD8 ratios and elevated CD4 T cell activation despite treatment. This indicates that while memory pTfh frequencies recover with antiretrovirals, these cells may still be qualitatively defective, with high PD-1 acting as a potentially pathogenic marker, as has been suggested by previous studies on HIV-specific CD4 (38) and CD8 (39) T cells. Alternatively a portion of PD-1+ pTfh cells may be Tfr cells with suppressive functions that account for low pTfh cell frequencies (40). Elevated PD-1 expression on pTfh cells correlated with increasing CD38+ HLA-DR+ CD4 and CD8 T cells, suggesting PD-1+ pTfh cells may be activated Tfh cells associated with inflammation in the adaptive immune system. Interestingly, Pallikkuth et al. reported that CD38+ HLA-DR+ expression on pTfh cells decreased after 48 weeks of treatment, but was still significantly higher than healthy controls (21). This activated pTfh cell state likely contributes to their susceptibility as a target for HIV infection and may be linked to high PD-1 expression.

T follicular helper cells also localize in the Peyer’s patches of the small intestine. Because there are significant disruptions to the intestinal mucosa during HIV infection, we examined the relationship between Tfh cells and gut-homing receptor α_4_β_7_ and I-FABP (41). It was previously shown that I-FABP is increased in perinatally HIV-infected children (42). We found that low memory pTfh cell frequencies in HIV+ subjects correlated with higher plasma I-FABP and lower β7+ memory CD4 T cell levels. One explanation may be that with worsening gut mucosal disruption, pTfh cells are trafficked to the gut. Localization to the gut may also account for lower circulating pTfh cells during HIV infection. Although we did not co-stain for CXCR5 and β7 together, pTfh cells likely co-express β7 to mediate homing to the intestine.

It has been well documented that memory B cell populations and the quality of B cell responses are substantially impaired in HIV+ adults and children (43). More recent reports demonstrate that inadequate B cell help by HIV-infected Tfh cells results in perturbed B cell differentiation and dysregulated antibody production (16, 17). In addition, dysfunctional Tfh cells activate non-specific B cells and lead to hypergammaglobulinemia characteristic of HIV infection (34, 40). Prior groups have shown decreased B_N_ and B_RM_ cell subsets, as well as expanded B_AM_ and B_TLM_ B cell subsets in HIV+ children (18–20). In our study, we had similar findings—total B cells were diminished and IgD+ B cells were elevated in HIV-infected children compared with healthy controls. We also demonstrated that B_N_ were increased in ART+, B_RM_ were low in HIV+, and B_AM_ and B_TLM_ were high in HIV+ compared with healthy controls. While pTfh cell proportions were significantly decreased in the blood, it is possible that with the accumulation of Tfh cells in the LNs, overstimulation by GC Tfh cells in the B cell follicles leads to a shift toward a more differentiated and exhausted B cell state. Moreover, lower total B cells and elevated IgD frequencies in HIV+ children suggest a general insult to the B cell compartment and a defect in Tfh cell function to induce class-switching in memory B cells. Decreased pTfh cells with potentially impaired B cell function may preclude effective HIV antibody responses. Indeed, prior studies report preserved pTfh cells in subjects with broadly neutralizing HIV-specific antibody responses (15, 44). Low pTfh cell frequency was closely linked to a differentiated B cell state in HIV+ children but not when separated into ART− and ART+ groups, suggesting antiretroviral treatment may restore the balance of Tfh cells with B cell differentiation.

Finally, Tfc cells are a novel CD8 T cell subset expressing CXCR5. Tfc cells have been studied in the blood and LN of SIV+ primate models (45, 46), the LNs of LCMV mouse models (33), as well as the blood, tumors, and LN of adult humans (32, 33, 47–49). Multiple groups reported Tfc cells have similar B cell follicle homing abilities to Tfh cells, and the potential to control viral infection by eliminating infected T cells and B cells in the follicle. He et al. reported that HIV-specific Tfc cells were present in the blood of chronically infected adults, and pTfc cells inversely correlated with HIV viral load prior to ART (33). More recently, Jiao et al. reported HIV+ adults had increased CXCR5+ CD8 T cells with high PD-1 expression, which negatively correlated with HIV disease progression (47). To the best of our knowledge, pTfc cell populations in children were not previously studied. HIV+ children had irreversibly depleted CXCR5+ CD8 T_M_ cells. Notably, antiretroviral treatment raised memory pTfc cell levels but failed to normalize them. As such, pTfc cells directly correlated with %CD4 and CD4:CD8 ratios regardless of treatment status. PD-1 expression was elevated on memory pTfc cells in HIV+ children and negatively correlated with CD4:CD8 ratios and positively associated with CD38+HLA-DR+ CD8 T cells. Interestingly, whereas Jiao et al. concluded CXCR5+ CD8 T cells with high PD-1 expression were highly functional and associated negatively with disease progression, our data demonstrated an association with CD4:CD8 ratios, but not with viremia or CD4 percentages. Our opposing findings may reflect differences in a pediatric cohort or our separate gating strategy on CD8 memory rather than total CD8 T cells.

In conclusion, we demonstrated a marked decrease in peripheral memory Tfh frequencies in untreated children with perinatal HIV infection. These memory pTfh cells increased with antiretroviral therapy but failed to normalize within 1 year. Treatment initiation at younger ages predicted greater recovery of this population. HIV+ children have high PD-1 expression on memory pTfh cells regardless of treatment status. Low memory pTfh cell frequencies with high PD-1 levels correlate with worsening HIV disease status as well as innate and adaptive immune activation. Furthermore, B cell subpopulations are skewed toward a differentiated and exhausted profile, and coincide with decreased memory pTfh cells in HIV+ children. Finally, memory pTfc cells are depleted in HIV+ children. This perturbed memory pTfh cell population may contribute to weak vaccine and HIV-specific antibody responses in HIV+ children. Together, these findings have important implications for ongoing pediatric HIV cure and vaccine strategies.

## Ethics Statement

This study was carried out in accordance with the recommendations of New York University Institutional Review Board and Kenyatta National Hospital/University of Nairobi Ethical Review Committee. The protocol was approved by the New York University Institutional Review Board and Kenyatta National Hospital/University of Nairobi Ethical Review Committee. All subjects gave written informed consent in accordance with the Declaration of Helsinki.

## Author Contributions

BM analyzed flow cytometry data, drafted the manuscript and figures. MM collected and processed samples and managed data. FM recruited patients and recorded clinical data. AA provided input to study design and oversaw recruitment site. AKravietz and TI performed immune phenotyping studies. MG performed sCD163 ELISA assay. PA performed I-FABP ELISA assay. AKhaitan, WB, and DU conceptualized the study, designed experiments, and interpreted data. AKhaitan supervised the study and edited the manuscript.

## Conflict of Interest Statement

The authors declare that the research was conducted in the absence of any commercial or financial relationships that could be construed as a potential conflict of interest.

## Supplementary Material

Figure S1 Peripheral Tfh (pTfh) cell correlations with %CD4, CD4:CD8 ratios, and immune activation in HIV negative children. Correlations between memory pTfh cells and **(A)** %CD4 and CD4:CD8 ratios, **(B)** CD38+ HLA-DR+ CD4 and CD8 T cells, and **(C)** plasma sCD163 levels in HIV negative children.

Figure S2 PD-1+ memory peripheral Tfh (pTfh) cell gating and correlations in HIV negative children. **(A)** FACS plots showing representative gating of PD-1+ memory pTfh cells in an HIV− and HIV+ subject. Plots shown were gated within the CXCR5+ CCR7+ CD4+ T_M_ population. Correlations are shown between PD-1+ memory pTfh cells and **(B)** %CD4 and CD4:CD8 ratios, **(C)** CD38+ HLA-DR+ CD4 and CD8 T cells, and **(D)** plasma sCD163 levels in HIV negative children.

Figure S3 Correlations between memory peripheral Tfh (pTfh) cells and B cell subsets in HIV-negative children. Correlations between total and PD-1+ memory pTfh cells and **(A)** CD27−CD21+ naïve (B_N_), **(B)** CD27+ CD21+ resting memory (B_RM_), **(C)** CD27+ CD21− activated memory (B_AM_), and **(D)** CD27−CD21− tissue-like memory (B_TLM_) B cell subsets in HIV negative children.

Figure S4 CXCR5+ memory CD8 T cell subsets and correlations with T cell activation. **(A)** Correlations between CXCR5+ CD8 T_M_ cell frequencies and %CD4 and CD4:CD8 ratios in HIV− children. Correlations between **(B)** total and **(C)** PD-1+ CXCR5+ CD8 T_M_ cell frequencies and CD38+ HLA-DR+ CD4 and CD8 T cells and plasma sCD163 levels in HIV− and HIV+ children (ART− in orange and ART+ in blue).
