# Supplementary figures and images for: Low Peripheral T Follicular Helper Cells in Perinatally HIV-Infected Children Correlate With Advancing HIV Disease
Source: Front Immunol. 2018 Aug 24;9:1901. doi: 10.3389/fimmu.2018.01901 (PMC6117426; doi:10.3389/fimmu.2018.01901)

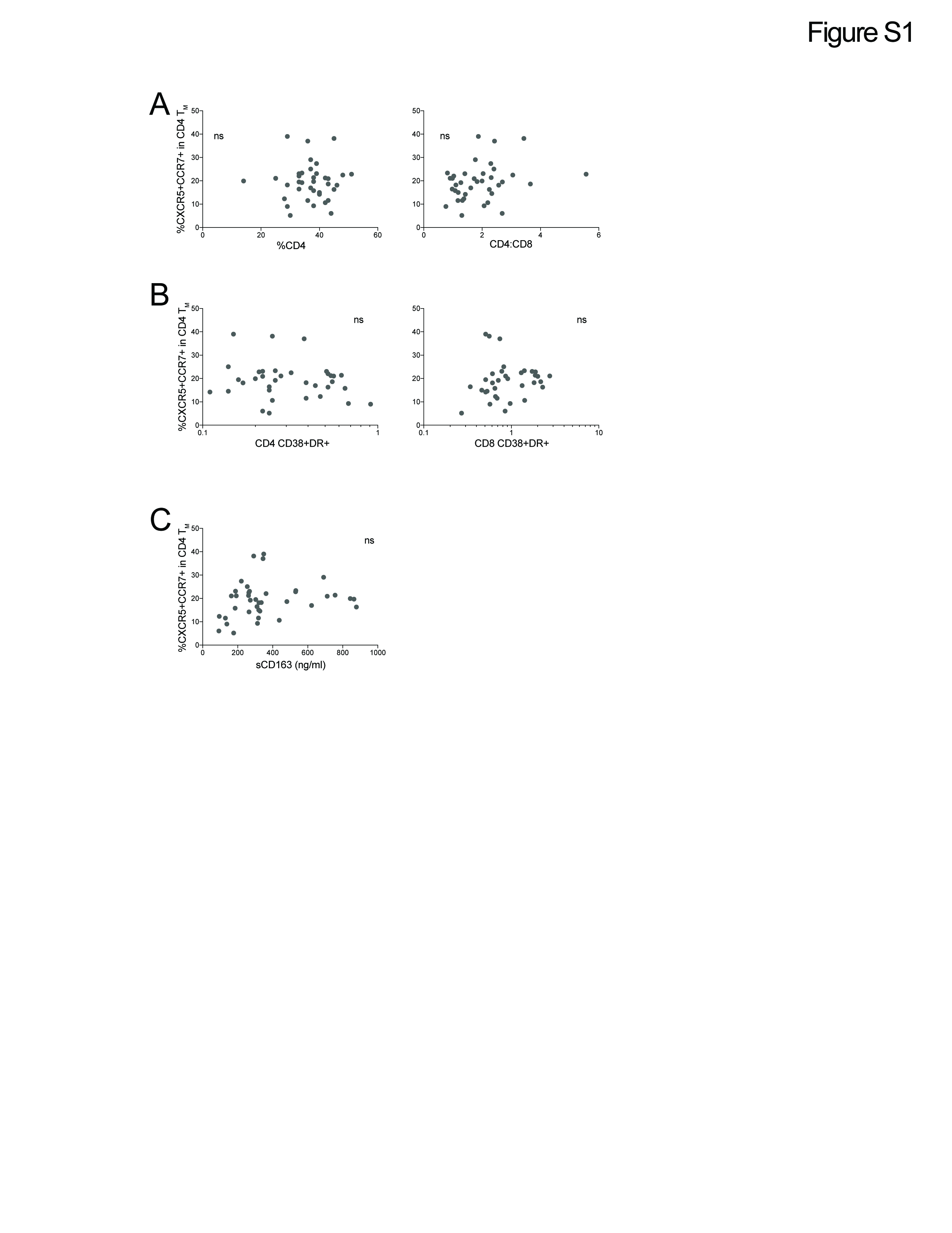

Supplement: Figure S1 — Peripheral Tfh (pTfh) cell correlations with %CD4, CD4:CD8 ratios, and immune activation in HIV negative children. Correlations between memory pTfh cells and (A) %CD4 and CD4:CD8 ratios, (B) CD38+ HLA-DR+ CD4 and CD8 T cells, and (C) plasma sCD163 levels in HIV negative children. [file image_1.tif]

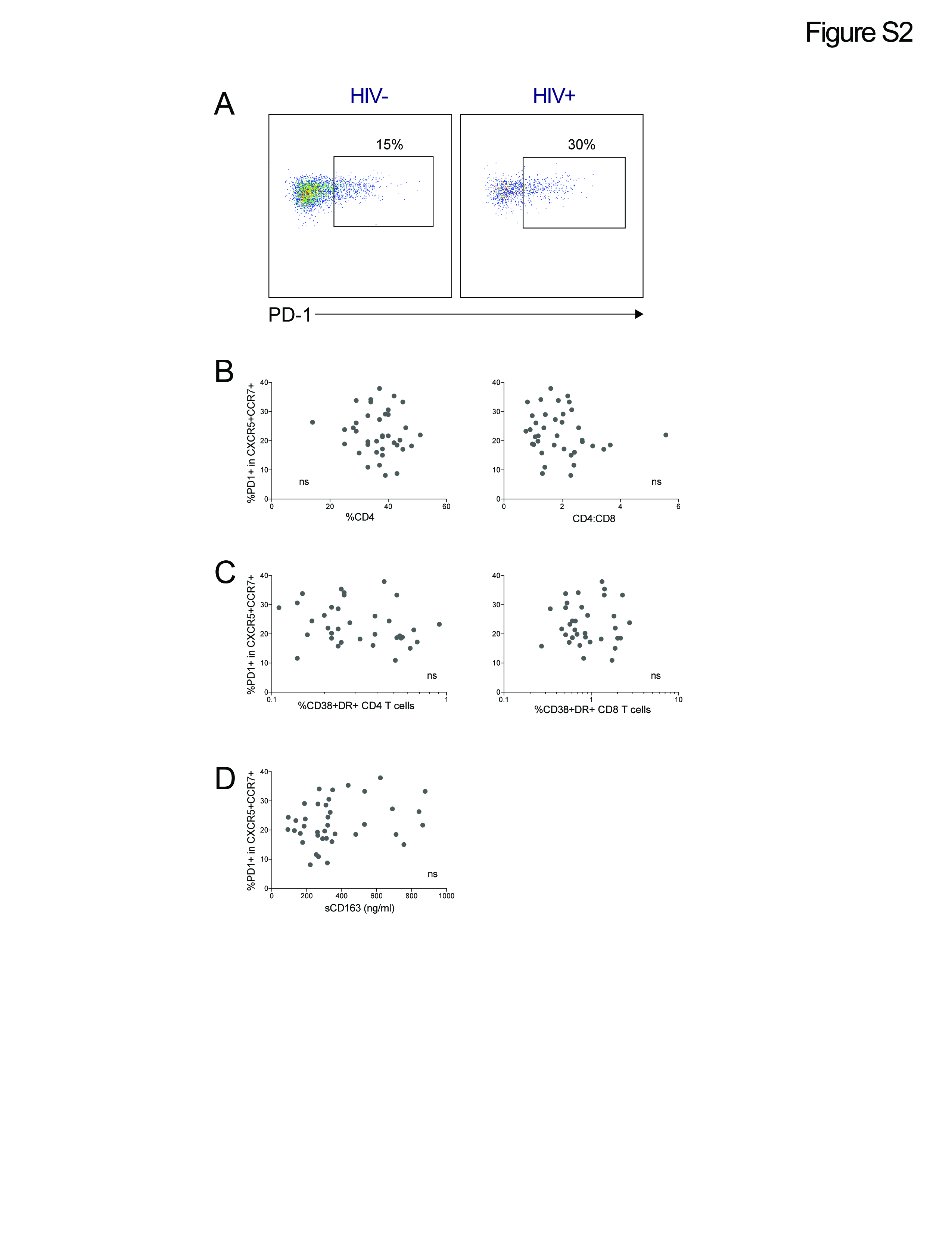

Supplement: Figure S2 — PD-1+ memory peripheral Tfh (pTfh) cell gating and correlations in HIV negative children. (A) FACS plots showing representative gating of PD-1+ memory pTfh cells in an HIV− and HIV+ subject. Plots shown were gated within the CXCR5+ CCR7+ CD4+ TM population. Correlations are shown between PD-1+ memory pTfh cells and (B) %CD4 and CD4:CD8 ratios, (C) CD38+ HLA-DR+ CD4 and CD8 T cells, and (D) plasma sCD163 levels in HIV negative children. [file image_2.tif]

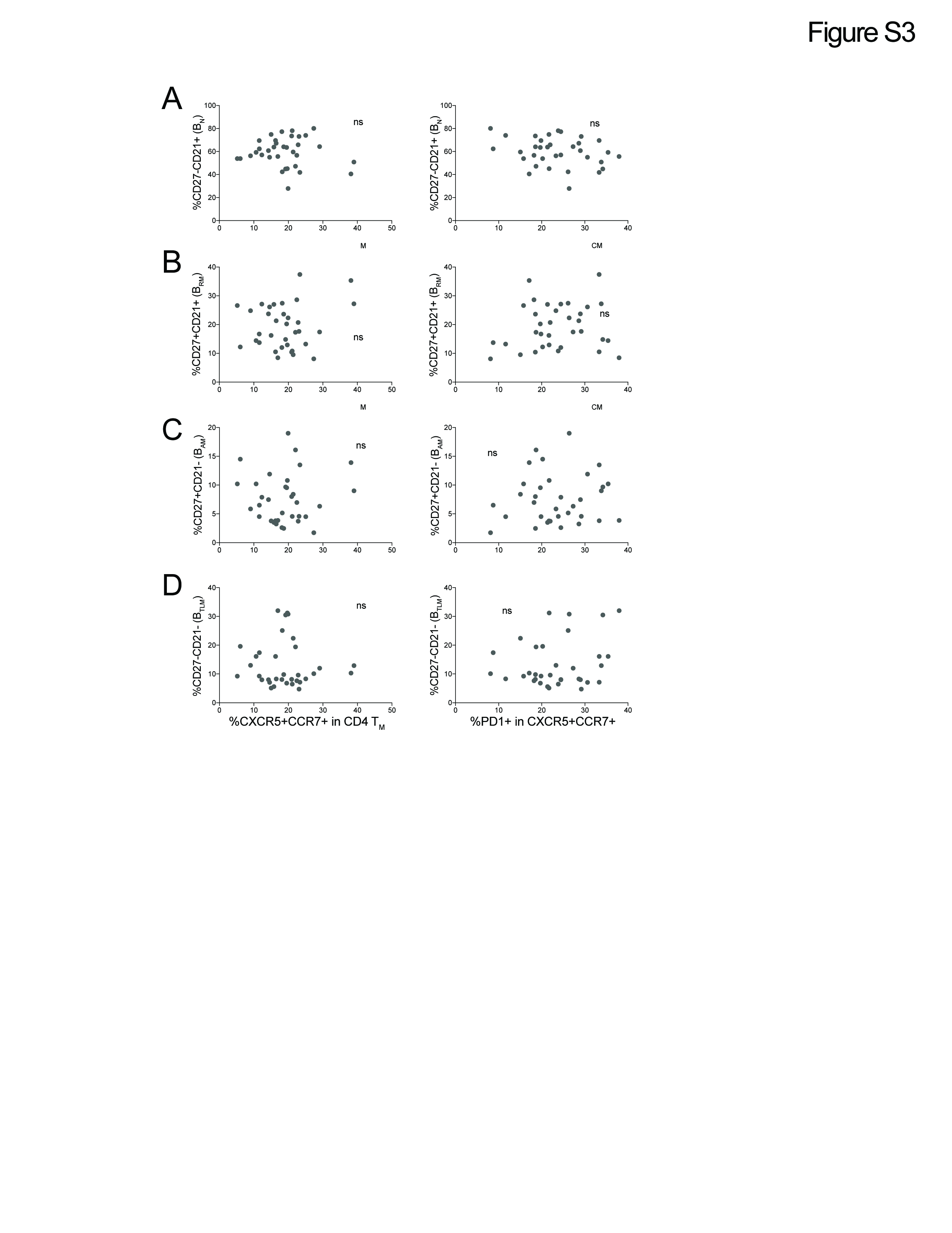

Supplement: Figure S3 — Correlations between memory peripheral Tfh (pTfh) cells and B cell subsets in HIV-negative children. Correlations between total and PD-1+ memory pTfh cells and (A) CD27−CD21+ naïve (BN), (B) CD27+ CD21+ resting memory (BRM), (C) CD27+ CD21− activated memory (BAM), and (D) CD27−CD21− tissue-like memory (BTLM) B cell subsets in HIV negative children. [file image_3.tif]

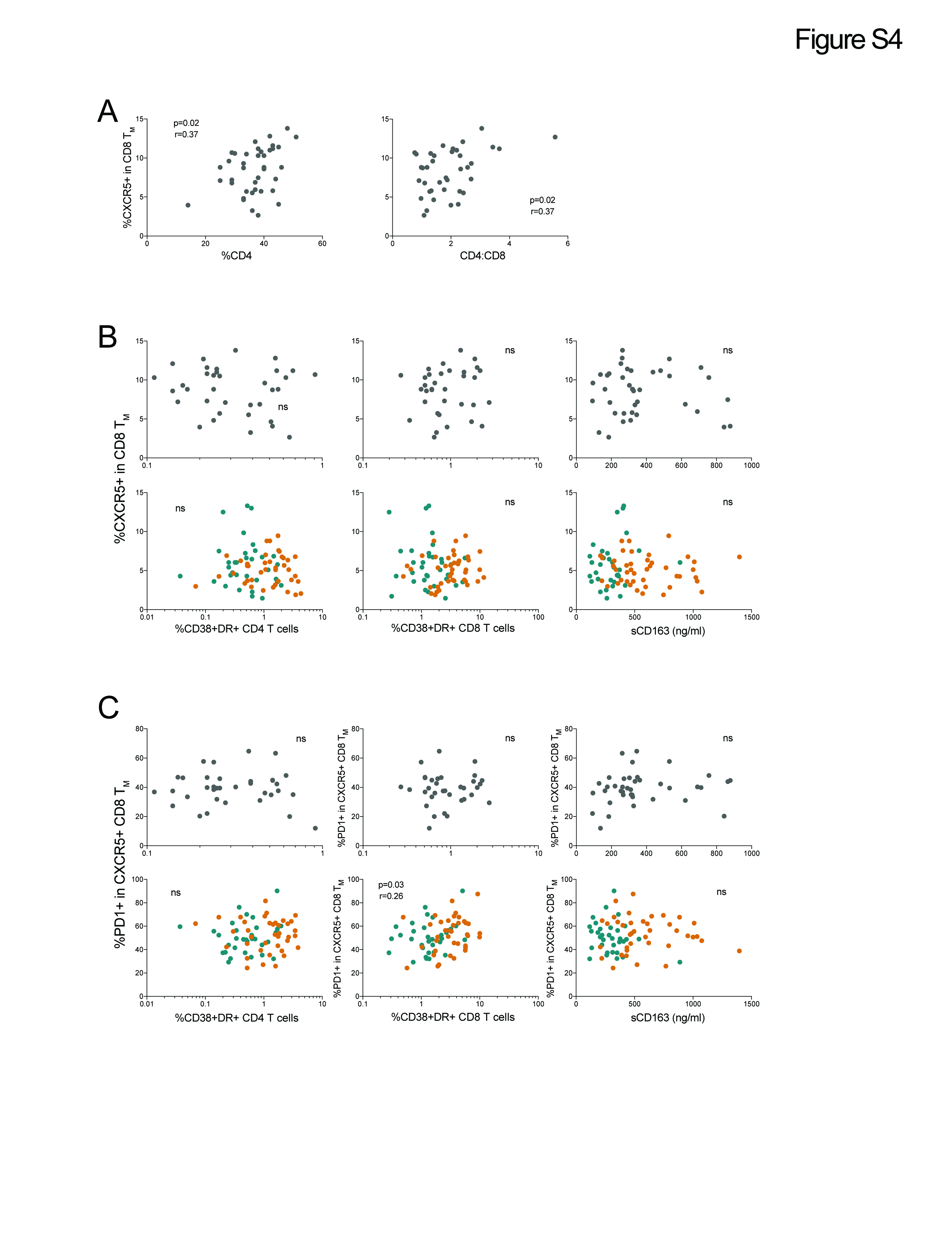

Supplement: Figure S4 — CXCR5+ memory CD8 T cell subsets and correlations with T cell activation. (A) Correlations between CXCR5+ CD8 TM cell frequencies and %CD4 and CD4:CD8 ratios in HIV− children. Correlations between (B) total and (C) PD-1+ CXCR5+ CD8 TM cell frequencies and CD38+ HLA-DR+ CD4 and CD8 T cells and plasma sCD163 levels in HIV− and HIV+ children (ART− in orange and ART+ in blue). [file image_4.tif]
