# Supplementary material for: Low Peripheral T Follicular Helper Cells in Perinatally HIV-Infected Children Correlate With Advancing HIV Disease
Source: Front Immunol. 2018 Aug 24;9:1901. doi: 10.3389/fimmu.2018.01901 (PMC6117426; doi:10.3389/fimmu.2018.01901)
Supplement: Supplementary file 5 [file data_sheet_1.PDF]

Table S1: Antiretroviral Timing in ART+ Subjects

PID	Age at ART Initiation (Years)	Years on ART
3	2.3	7.3
4	10.0	2.7
7	0.8	5.5
8	9.7	3.5
9	1.1	8.9
13	8.5	4.6
22	10.5	2.4
23	7.1	0.5
27	2.8	2.6
30	4.7	5.1
33	8.2	1.0
34	2.0	5.6
35	5.3	1.3
46	5.5	5.2
49	7.1	2.0
50	3.7	4.8
51	1.4	4.4
52	11.6	1.7
53	9.0	6.8
56	6.1	2.6
60	11.0	1.0
61	5.9	1.0
73	10.3	1.5
74	9.6	3.7
76	6.7	2.6
86	13.9	4.1
120	9.2	4.9
130	11.6	6.4
132	11.5	2.3
133	12.5	0.5
136	5.3	6.6
138	12.5	2.6
139	8.8	3.9
**Median (IQR)**	**8.2 (5.0-10.4)**	**3.5 (1.8-5.2)**
