# Supplementary material for: Low Peripheral T Follicular Helper Cells in Perinatally HIV-Infected Children Correlate With Advancing HIV Disease
Source: Front Immunol. 2018 Aug 24;9:1901. doi: 10.3389/fimmu.2018.01901 (PMC6117426; doi:10.3389/fimmu.2018.01901)
Supplement: Supplementary file 6 [file data_sheet_2.PDF]

Table S2: Multivariate regression models predicting CXCR5+ T cell subsets based on HIV status and age

	Coefficient (standard error)	p value
CXCR5+CCR7+ in CD4 T_M	21.4 (3.4)	6.99E-09
CXCR5 in CD8 T_M	11.3 (3.6)	0.002
PD-1+ in CXCR5+CCR7+ in CD4 T_M	13.7 (4.5)	0.003
PD-1+ in CXCR5+ CD8 T_M	37.7 (5.9)	4.17E-09

In a multiple linear regression analysis using R programming, each CXCR5+ T cell subset was predicted by HIV status when including age as a covariate
